# Research Progress of Hyperfluorescent Organic Electroluminescent Devices

**DOI:** 10.3390/mi17010040

**Published:** 2025-12-29

**Authors:** Yaxin Li, Jiaqi Wang, Chaoteng Pan, Xin Jiang, He Dong, Jin Wang, Gang Zhang

**Affiliations:** 1 College of Information Technology, Jilin Engineering Research Center of Optoelectronic Materials and Devices, Jilin Normal University, Siping 136000, Chinazg982@163.com (G.Z.); 2 Jilin Provincial Key Laboratory of Wide Bandgap Semiconductor Material Growth and Device Applications, Jilin Normal University, Changchun 130103, China; 3 State Key Laboratory of Integrated Optoelectronics, College of Electronic Science and Engineering, Jilin University, Changchun 130012, China

**Keywords:** hyperfluorescent organic light-emitting diodes (HF-OLEDs), light-emitting materials, light-emitting mechanism, TADF

## Abstract

Organic light-emitting diodes (OLEDs) have the advantages of high efficiency and high color purity, which gives them great potential and application prospects in the field of display technology, and thus they have been of wide interest for scholars and industry. Due to their nature, when using the first generation of fluorescent materials, only 25% of the excitons are used, while the rest are wasted, meaning the device efficiency does not exceed 25%. The second generation of phosphorescent materials solves this problem by utilizing 25% singlet excitons while utilizing 75% triplet excitons, achieving 100% internal quantum efficiency. Therefore, a third generation of materials, namely Thermally Activated Delayed Fluorescence (TADF) materials, has been developed, and these are able to use the small singlet–triplet energy gap to allow excitons on the triplet state to upconvert back to the single state, which improves the utilization of triplet excitons. These TADF materials can also reach 100% maximum internal quantum efficiency, but they have many problems, such as low color purity and serious efficiency roll-off. Therefore, researchers have designed hyperfluorescent materials, which possess high efficiency, high color purity, and a long lifetime, showing tremendous potential and application prospects in the field of display technology. This report takes hyperfluorescent OLEDs as the entry point and the molecular design and luminescence mechanism of hyperfluorescent materials are reviewed, considering blue, green, red, and white light.

## 1. Introduction

Fluorescent materials follow the spin conservation law in the process of luminescence. These materials are not only rich in variety and economical in price but also have high photoluminescent efficiency. However, a major limitation of fluorescent materials is that they can only use singlet excitons for luminescence, rather than using trilinear excitons directly. As a result, trilinear excitons are eventually converted to heat energy through non-radiative transitions and dissipated. This inefficiency results in significant energy loss, which affects the overall performance of OLED devices.

In 1998, Professor Forrest’s team at Princeton University successfully synthesized room-temperature phosphorescent materials by introducing heavy-metal atoms into organic groups [1]. This significant discovery significantly improved the phosphorescent OLEDs’ internal quant efficiency to reach 100% [2]. However, with phosphorescent OLEDs forming an important system of light-emitting materials, the length of their lifetime has unexpectedly become the main factor to consider due to the fact that the efficiency roll-off phenomenon is particularly serious under conditions that involve a high-current injection. Maintaining the main material in a high-energy state for a long time will accelerate the aging of the device and affect its service life. In addition, the design cost is also an important issue, and these factors together limit the commercial application of OLEDs.

In 2010, the Adachi team made an important scientific achievement. They succeeded in designing a new TADF material [3]. These TADF materials have a small ΔE_ST_, which reduces energy waste. Therefore, TADF also exhibits good charge transfer (CT) properties [4]. However, TADF materials have a large full width at half maximum, which makes them unusable in applications requiring high color purity. At the same time, TADF materials have a long exciton lifetime, which can lead to material degradation and a corresponding reduction in efficiency during long-term use, especially in regions of high-brightness emissions [5]. We found that TADF materials achieve high electroluminescence efficiency, but that it is difficult to maintain good color purity and purity requirements when using them.

**Figure 1:**
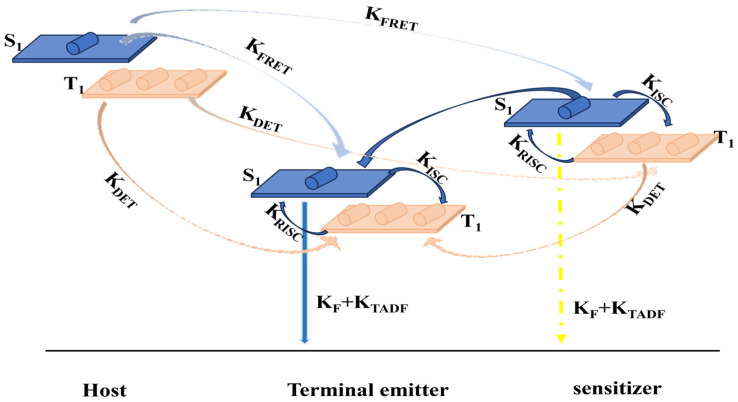
Schematic diagram for the hyperfluorescent process [14].

In order to cope with the various challenges faced by TADF devices, Zhang Dongdong’s research group [6] proposed a new TADF device structure, namely the hyperfluorescent system [7]. This technology allows OLEDs to achieve a high level of color purity, and this technology also achieves 100% exciton utilization efficiency [5,8]. Their new device uses a TADF material and a fluorescent material as the main materials to form the luminescent layer, to maximize the advantages of TADF materials. Among them, the TADF material acts as a sensitizer to transfer energy to the fluorescent luminescent material and make it glow, so that 100% exciton utilization is achieved and a higher color purity is obtained through the fluorescent luminescent material. The core mechanism involves transferring energy from the TADF material to the fluorescent material through Förster Energy Transfer (FRET), which enables the fluorescent light-emitting material to obtain more singlet excitons from the TADF material, thus producing pure luminescence. This FRET process can improve the performance of OLEDs while shortening the exciton lifetime of the TADF material [9,10,11,12,13,]. This high-frequency technology can improve the efficiency, durability, and purity of the device’s colors. The energy transfer process of this hyperfluorescent system is shown in Figure 1 [14]. OLEDs select a TADF molecule as the sensitizer and emit light from the fluorescent material. The TADF material can upconvert triplet excitons to singlet excitons, then transfer exciton energy to the fluorescent material via FRET, and then light is emitted due to the radiative transition of the fluorescent material.

TADF sensitizes the fluorescence mechanism due to the decomposition of the upconverted excitons’ function and the radiative transition in the luminescent layer. Combining the advantages of the TADF molecules’ fast upconversion and the luminescent material molecules’ fast radiative transition leads to the achievement of a longer life for the device. At the same time, the fluorescent material can be selected with a narrow spectrum of materials, which improves the color purity of the device, allowing a more excellent device to be obtained [15].

## 2. Development of Hyperfluorescent Organic Light-Emitting Diodes

Hyperfluorescent OLEDs have emerged as an advanced emissive technology that enables efficient utilization of triplet excitons through synergistic light-emission mechanisms, thereby achieving simultaneously high efficiency, narrow emission bandwidth, and extended operational lifetime.

In the development of hyperfluorescent OLEDs, researchers primarily focused on enhancing the overall efficiency of OLED devices. However, conventional OLEDs suffered from several inherent limitations, including low color purity and short operational lifetime. Early OLEDs mainly employed traditional fluorescent materials, in which only singlet excitons contribute to light emissions. The triplet excitons, possessing relatively long lifetimes, tend to accumulate and induce material degradation, ultimately leading to reduced device efficiency and stability. To overcome these challenges, researchers began to explore alternative luminescence mechanisms, which eventually led to the emergence of hyperfluorescent technology.

The introduction of hyperfluorescent technology marks a significant technological breakthrough. In this device architecture, the TADF material is incorporated as an assistant dopant within the host matrix, while light emission is ultimately produced by a fluorescent emitter. Researchers at Kyushu University successfully demonstrated a hyperfluorescent OLED with an external quantum efficiency (EQE) of 32% and an emission bandwidth of only 19 nm, indicating exceptionally high color purity and excellent device stability. Subsequent studies further optimized the device architecture and material design, leading to hyperfluorescent OLEDs with superior performance.

The development of hyperfluorescent OLED technology exemplifies the power of continuous technological innovation. Ongoing research and experimental efforts are steadily advancing this technology, contributing to significant improvements in display and lighting applications. With deeper investigation and further technological progress, hyperfluorescent OLEDs are expected to demonstrate their unique advantages and broad potential across an even wider range of applications.

### 2.1. Blue Hyperfluorescent OLEDs Based on TADF-Sensitized Fluorescence

OLEDs are widely regarded as one of the most promising light-emitting technologies [16,17]. Despite substantial advances in this field, blue OLEDs continue to face significant challenges, particularly in achieving high color purity and overall device performance. The development of emissive materials with intrinsically narrow emission spectra remains highly demanding in terms of both material design and resource investment. As this challenge has not yet been fully overcome, conventional blue fluorescent materials are still extensively employed in commercial OLED applications.

Faced with the challenges associated with traditional OLED technology, scientists have innovatively designed a hyperfluorescent mechanism that effectively harvests high-energy triplet excitons through the triplet–triplet fusion (TTF) process, thereby preventing their accumulation in blue OLED devices. This strategy shortens the effective exciton lifetime, reduces exciton annihilation, and significantly improves the overall stability and efficiency of the devices.

**Figure 2:**
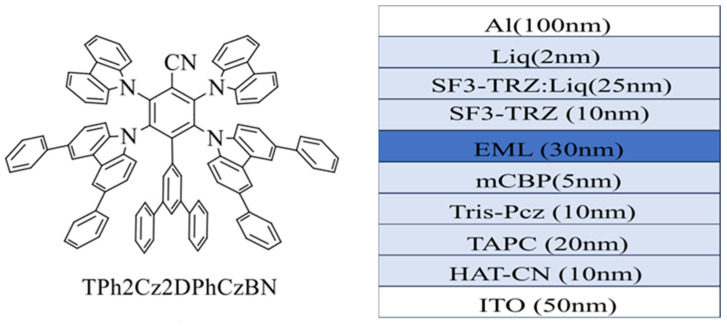
The molecular formulas of the main materials used in the experiment and the device structure of the fabricated devices [18].

In 2021, Chan et al. [18] synthesized a new material TPh_2_Cz_2_DPhCzBN [18]. The bulky *m-*terphenyl groups effectively suppress molecular aggregation in thin film; thereby, maintaining high color purity in the device. In addition, the rigid molecular structure inhibits bimolecular interactions, which is expected to enhance device stability. Owing to these properties, TPh_2_Cz_2_DPhCzBN can be employed as a sensitizer in hyperfluorescent OLED systems. The molecular formulas of the key materials used in the experiment, as well as the device structure of the fabricated OLEDs, are shown in Figure 2 [18]. Due to the c donor and acceptor configuration of the TADF material, triplet excitons can be efficiently upconverted and transferred to a pure-blue fluorescent emitter, even when the TADF sensitizer itself exhibits sky-blue emissions. This energy transfer is realized through thermally activated processes, thereby significantly enhancing the emission performance of the pure-blue fluorescent emitter ν-DABNA.

The synthesized TPh_2_Cz_2_DPhCzBN is a TADF material with optoelectronic properties. Typically, TADF materials exhibit relatively broad electroluminescence (EL) spectrum, which is undesirable for practical display applications. Therefore, this experiment adopts a new hyperfluorescent technology to solve this problem. A TADF material and a narrow emission fluorescent material are added to the host material as a luminescent layer [5]. In this work, TPh_2_Cz_2_DPhCzBN was used as TADF material, while ν-DABNA was used as the fluorescent emitter [15]. Since the emission spectrum of TPh_2_Cz_2_DPhCzBN overlaps significantly with the absorption spectrum of v-DABNA, energy can be transferred to the fluorescent material to a large extent and used for luminescence. As a result, narrowband blue emission is obtained, leading to a substantial improvement in the color purity of the device.

**Figure 3:**
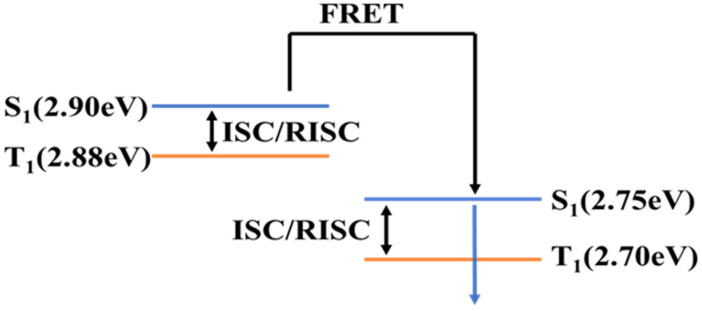
Blue hyperfluorescent OLED energy transfer process [18].

In this hyperfluorescent system, singlet excitons generated in the host material mCBP transfer energy to the singlet states of TADF material TPh_2_Cz_2_DPhCzBN and the fluorescent emitter v-DABNA via the FRET. In addition, triplet excitons in the TADF material are upconverted to singlet excitons through the reverse intersystem crossing (RISC) process, then continue to transfer energy to the fluorescent material, and finally more energy is transferred to the fluorescent emitter. Consequently, a larger proportion of excitons contribute to light emission from ν-DABNA, resulting in narrowband blue emission. The energy-transfer processes are illustrated in Figure 3 [18].

The concept demonstrated in this study enabled the successful fabrication of OLEDs with excellent performance. The devices achieved a maximum EQE of 27%, and a narrow full width at half maximum (FWHM) of only 18 nm, indicating high color purity. These results confirm the feasibility of the hyperfluorescent device architecture. Moreover, the hyperfluorescent structure exhibits a shortened triplet exciton lifetime, which facilitates faster triplet exciton consumption, suppresses efficiency roll-off, and maintains narrowband emission.

In the hyperfluorescent OLED, TPh_2_Cz_2_DPhCzBN improves the emission color through the FRET process. This kind of OLED, which primarily generates singlet excitons, exhibits excellent stability under pure-blue emission. In contrast, when ν-DABNA is used alone as the emissive layer, the device lifetime is relatively short due to the instability of the triplet state. The device designed in the experiment has the advantages of narrow emission, well color purity and long-term operation. Meanwhile, the devices prepared in this experiment have significantly longer service life compared to the OLEDs previously fabricated based on ν-DABNA. Due to the larger K_RISC_ of the prepared materials, this helps to consume triplet excitons more quickly than v-DABNA. Thus, the device has higher stability, and the incorporation of more stable TADF sensitizers, the device’s lifespan also increases. This structure also makes the device more suitable for commercial use.

In hyperfluorescent OLEDs, Dexter energy transfer (DET) also occurs, which constitutes an energy loss process. This occurs when the molecular orbitals overlap and the distance between donor and acceptor is short [19,20]. An effective method to inhibit this process is to introduce inert clumps between the TADF material and the fluorescent material, reducing the molecular orbital overlap and increasing the distance between the donor receptor [21,22,23]. In 2018, Duan et al. attempted to introduce inert groups in the fluorescent material, changing the polarity of the molecule. This method reduces the DET process and improves the performance of the device [10]. Similarly, Su et al. also conducted experiments on this strategy, introduced inert groups into PXZ-DBPZ, and developed a new TADF material FPXZ-DBPZ [24]. The results show that the energy transfer process can be reduced through reasonable molecular design. At the same time, Lee et al. also discovered a new method. By speeding up the RISC process, more excitons are transported back to the singlet state and then transferred by the FRET process instead of being wasted [25]. This is also a good way to solve the problem of wasting energy in the DET process.

In 2023, Lee and his team conducted innovative experimental research on the selection of sensitizers [26]. They selected DBA-DmICz and DBA-DTMCz as sensitizers and prepared a hyperfluorescent OLED with the following structure: ITO (50 nm)/HATCN (7 nm)/TAPC (55 nm)/DCDPA (10 nm)/DBFPO:30% DBA-DmICz or 30% DBA-DTMCz:1% v-DABNA (25 nm)/DBFPO (10 nm)/TPBi (20 nm)/LiF (1.5 nm)/Al (100 nm) [26]. Through the test analysis, Lee et al. further verified that Dexter energy transfer process can lose energy in the hyperfluorescent system. In this experiment, Lee et al. conducted experiments on this finding and solved it. They increased the RISC process of TADF materials, so that more triplet excitons were upconverted into singlet excitons, thus reducing the DET process, which effectively reduced the energy loss. Therefore, the issue of energy loss associated with the Dexter energy transfer (DET) process is effectively mitigated. Reducing DET minimizes energy loss, thereby enabling the fabrication of more efficient hyperfluorescent OLED.

The blue hyperfluorescent OLEDs with the new architecture exhibit significantly improved the external quantum efficiency compared with traditional blue OLEDs and achieved narrowband emission with excellent color purity. In terms of stability, it has also been significantly improved, which means that the manufacturing process is further improved and its operational lifetime is expected to be significantly extended. Thanks to these excellent performance characteristics, blue hyperfluorescent OLEDs will show great application potential in the future. Meanwhile, the multi-resonance TADF materials used in the experiment have extremely narrow emission spectra and higher color purity, making them an ideal blue light material [14,27].

In 2024, Wang et al. [28] investigated the influence of isotope engineering on the relationship between intrinsic molecular stability and the operational stability of the devices [29]. This process mainly targeted the weakening of energy loss caused by C-H bond vibrations in Multiple-Resonance Thermally Activated Delayed Fluorescence (MR-TADF) materials [30]. This strategic deuterated method highlights the importance of material structure modification in improving the operational stability of the devices. Through the deuterated process, the photostability of the deuterated compounds was also enhanced. This plays a crucial role in extending the lifespan of the devices. By simultaneously introducing deuterated dibenzofuran units and deuterated phenyl parts, the lifespan of the LT80 device was significantly prolonged. This study will provide valuable insights into extending the stable operating time of devices through modifying the chemical structure [31]. Although the blue hyperfluorescent OLED technology has demonstrated excellent performance, there are still some technical challenges in its commercialization process [32]. To ensure that this new OLED technology can be widely applied in the display and lighting markets, researchers and engineers are working hard to improve the shortcomings of this device [33]. The characteristics of the high-performance blue HF-OLEDs devices reported in recent years are shown in Table 1.

### 2.2. Green Hyperfluorescent OLEDs Based on TADF-Sensitized Fluorescence

Compared with fluorescent materials, TADF materials can utilize both singlet and triplet excitons, thereby enhancing the overall performance of the device [34,35,36,37,38,39,40]. In recent years, an increasing number of researchers have focused on the study of TADF materials, particularly TADF materials based on metal complexes. This TADF material is able to produce a shorter delayed fluorescence lifetime (τd) and a stronger spin–orbit coupling (SOC) effect due to its structure. These optimizations are often closely related to the atomic number of the metal used [41,42,43,44,45,46,47].

In green hyperfluorescent OLEDs, researchers continue to explore the structural combinations of this TADF-sensitized fluorescence, designing various configurations and conducting systematic experiments. Finally, it was found that only when TADF materials are properly matched with fluorescent materials, and the minimum exciton energy level difference between the two materials is not too small and not too large, is it possible to achieve higher color purity requirements and obtain higher EQE. In response to this discovery, Professor Wang Kai’s team at Soochow University proposed a new design strategy that uses highly twisted thick ring π-conjugated MR-TADF molecules [48]. Based on this strategy, they designed the DBTN-2 material, which not only has excellent color purity and high efficiency, but also achieves CIE color coordinates (0.19, 0.74), far exceeding the green primary color in commercial displays, and approaching the green light standard of Rec.2020.

In 2019, Thompson et al. reported several new TADF materials [41]. These Cu(I) complexes have excellent photophysical properties such as almost uniformly high photoluminescence quantum yield (PLQY) and short luminescent lifetimes of 2–3 μs. In the same year, Yang et al. developed this material based on Thompson as a sensitizer for MR-TADF material [43]. Due to its inherent strong SOC effect, this complex has a faster RISC rate, enabling faster upconversion of excitons to singlet state, thus reducing energy loss. Finally, a device consistent with the yellow hyperfluorescent OLED is obtained. By 2022, Che et al. designed a highly stable Cu(I)-TADF luminescent material for use in OLEDs, achieving EQE of 23.6% and device lifetime (LT90) lasting 1300 h at 1000 cd m^−2^ [44]. The luminescent material has a high radiative rate constant and the PLQY is 89%. In terms of other ubiquitous metal complexes, Adachi et al. designed many light metal complexes based on Li, Mg, Zn, and Al in 2015 [45]. With the exception of Al, these complexes are mononuclear. The reported Al complexes are binuclear and bridged by OH groups. Among them, the PLQY of the Zn-based TADF complex is 78%, τԁ is 37.8 μs, and EQE is 19.6%. In 2021, Hisahiro and Kido developed another TADF material for solution-based OLEDs [47]. Among these Al (III)-TADF emitters, a TADF material, Al(DMACPDO)_3_, has a similar spectral curve to 4CzIPN and is therefore a typical TADF material [49,50,51], making Al(DMACPDO)_3_ a promising sensitizer in the MR-TADF field, but these materials are not widely used in solution-based hyperfluorescent systems [18,51,52,53].

**Figure 4:**
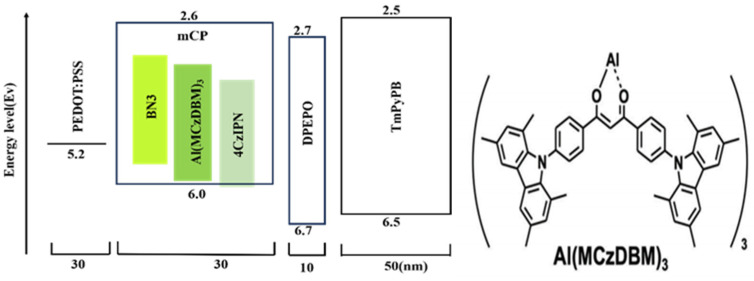
Molecular formula and device structure [54].

Although metal-based TADF complexes are generally considered the most promising in the OLED field, the number of such complexes that have been studied to date remains relatively small. Therefore, in 2024, Hisahiro et al. designed a new TADF material called Al(MCzDBM)_3_ [54], which is considered to be an excellent sensitizer for MR-TADF emitters. Its molecular formula and device structure are shown in Figure 4 [54].

When diluted in the host material mCP, mCP films doped with 5 wt.% Al(MCzDBM)_3_ exhibited green luminescence at 534 nm. In applications as sensitizers, a high PLQY at elevated doping concentrations is essential for the efficient transfer of Förster energy to the terminal luminescent material [18,51,52,53]. When the doping concentration reaches 50% wt.%, the PLQY of the film reaches 86%. Therefore, the Al(MCzDBM)_3_ significantly inhibited ACQ behavior compared to the representative TADF luminescence material 4CzIPN. Al(MCzDBM)_3_ exhibits a high k_r_ of 5.58 × 10^7^ s^−1^ and significant TADF properties in mCP films, where the ΔE_ST_ is only 0.11 eV and the τԁ is very short. It is 4.0 μs. The photophysical properties are significantly superior to those of the corresponding diketone ligands and are comparable to 4CzIPN. Natural orbital transitions and SOC calculations show [55] that metal complexation produces a high density Sm/Tn state with a larger SOC matrix element (SOCME) value than MCzDBM, which accelerates the RISC process.

The prepared material exhibits excellent photophysical properties, with a high PLQY of 97% and suppressed ACQ effects. The prepared material is used as the TADF material and BN3 as the fluorescent material. Compared with 4CzIPN, the prepared Al(MCzDBM)_3_ material has a larger spectral overlap with BN3 material, so its FRET process is better than 4CzIPN, which can faster upconvert triplet excitons into single excitons, reducing energy loss, so as to obtain devices with more excellent performance. Therefore, the researchers’ new attempt has once again confirmed that TADF materials based on metal complexes not only have good TADF properties but also can speed up the FRET process and reduce energy waste to obtain more efficient devices. In this experiment, a high emission Al (III)-TADF complex—Al (MCzDBM)_3_ with three *β*-diketone ligands—was developed and used as an effective sensitizer for the MR-TADF emitter. This complex exhibits excellent photophysical properties, with a high PLQY of 97% and a reduced ACQ. which is comparable to that of the representative green TADF emitter 4CzIPN. The final device’s EQE reaches 23.6%. This performance is significantly superior to that of the corresponding β-diketone ligand. The Al complex allows for the sensitization of the yellow MR-TADF emitter BN3, resulting in a nearly uniform PLQY and achieving an OLED with an EQE of 21.6% and a FWHM of 45 nm. The performance also exceeds that of devices based on 4czipn. This research opens up new avenues for the development of OLED technology. And the characteristics of the high-performance green HF-OLEDs devices reported in recent years are shown in Table 2.

Although green hyperfluorescent OLEDs exhibit excellent performance, efficiency roll-off at high luminance remains a challenge. To meet the stringent requirements for green color purity in Ultra HD display, researchers have developed a number of new MR-TADF materials [54,56]. By limiting the molecular relaxation process, these materials achieve narrow band luminescence with the FWHM close to that of inorganic materials.

With continued technological advancements and performance improvements, green hyperfluorescent OLEDs are expected to be applied in a wider range of display and lighting applications. From high-end mobile phone screens to flat panel displays to energy-efficient lighting systems, this technology shows great potential. Green hyperfluorescent OLEDs, with their innovative material combinations and molecular design strategies, have a great improvement in the efficiency and color purity of display technology. Although the hyperfluorescent OLED technology improves the life of blue OLEDs, the lifetime problem of the green OLED devices has not been improved, which is mainly due to the photothermal degradation of organic materials and the accumulation of triplet excitons. Therefore, although the green hyperfluorescent OLEDs have significant advantages in many aspects, they still face many challenges in terms of cost, life, and so on. However, with the continuous deepening of research and the development of new materials, green hyperfluorescent OLEDs will receive greater attention and be widely used.

**Table 2:** The characteristics of the reported high-performance green HF-OLEDs devices.

Emitters	CE (cd A^−1^) ^a^	PE (lm W^−1^) ^b^	EQE (%) ^c^	FWHM (nm) ^d^	CIE ^d^	Ref
4CzIPN	69.6/28.4	61.2/13.5	19.1/7.8	56.0	(0.36,0.57)	[54]
Al(MCzDBM)_3_	83.4/66.8	71.6/15.8	21.6/9.3	45.0	(0.39,0.58)	[54]
TTPA	45.0/38.0	47.0/30.0	15.8/11.7	57.9	(0.29,0.59)	[5]
m-Cz-BNCz	57.4/27.7	53.7/12.8	15.7/7.1	38.0	(0.28,0.65)	[57]
PhtBuPAD	72.2/61.8	59.7/35.9	21.2/18.1	45.0	(0.32,0.59)	[58]
4CzIPN	-/-	-/-	19.9/6.4	56.0	(0.39,0.59)	[40]

^a^ Current efficiency maximum value, value at 1000 cd m^−2^; ^b^ power efficiency maximum value, value at 1000 cd m^−2^; ^c^ EQE maximum value, EQE value at 1000 cd m^−2^; ^d^ CIE and FWHM at 1000 cd m^−2^.

### 2.3. Red Hyperfluorescent OLEDs Based on TADF-Sensitized Fluorescence

OLEDs have received considerable attention in large-area display devices and flexible screens. Although fluorescent and phosphorescent OLEDs have been successfully commercialized, their performance in aspects such as operational lifetime and production cost still requires further improvement.

In the development of display technology, there is a need to develop a new type of metal-free, highly efficient red OLED with high color purity and long operational lifetime. However, the red materials tend to have a low PLQY [59,60,61]. In addition, due to the wider emission spectrum of red materials, the color purity is not high [62,63]. Therefore, MR-TADF compounds are attractive in OLEDs because they show narrow-band emission, are bright, and can capture singlet and triplet excitons for light emissions. This is reflected in the fact that there are few examples of developing countries moving from orange to red emission countries [64,65]. However, a compound called boron dipyrromethene (BODIPY) is promising to solve these problems. Because of its special properties, high PLQY, and narrower FWHM compared to other red materials, OLED devices with higher color purity can be obtained when this material is applied as a fluorescent material to OLEDs [66,67,68]. However, the BODIPY material is essentially a fluorescent material, so when it is used as an OLED emission layer alone, its efficiency is very low, less than 5%, so the BODIPY material can be introduced into the hyperfluorescent structure, so that it can obtain better efficiency.

Duan et al. demonstrated that using BODEPY as a fluorescence dopant in a hyperfluorescent system results in higher EQE and improved device performance compared with using BODEPY as the emissive layer alone [69]. But in previous studies, the introduction of BODEPY materials into hyperfluorescent structures has not been much, and at the same time, there are no examples of the use of BODIPY materials in red HF-OLEDs to be found in the existing literature. Therefore, the data is not perfect. Therefore, the researchers show that further exploration of BODEPY materials is a new research direction. This requires deeper exploration of its molecular structure and internal energy levels, so that BODEPY materials can be more widely used in hyperfluorescent systems. At the same time, as with other colors of hyperfluorescent OLEDs, one of the reasons for its low efficiency is the Dexter energy transfer process, which is an energy loss process. To solve this problem, the researchers conducted a lot of experiments. Lee et al. found that the introduction of electron inert groups into TADF materials can effectively improve this problem, resulting in more efficient OLEDs [70]. At the same time, Duan et al. also found through research that the introduction of electron inert tert-butyl around fluorescent materials can also effectively improve this problem, and the resulting device efficiency roll-down is very low [10]. Although HF-OLED has shown good efficiency and service life, its overall performance still fails to surpass phosphorescent OLEDs.

**Figure 5:**
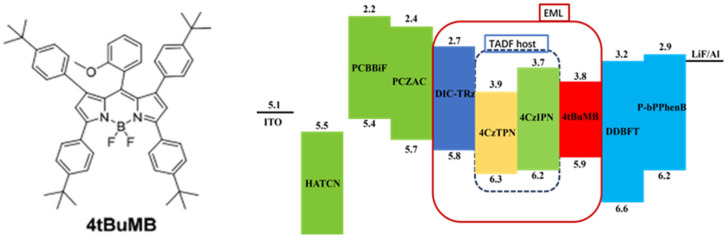
Molecular formula and device structure [23].

Therefore, in 2021, Lee et al. [23] designed a new hyperfluorescent architecture in which the BODEPY derivative 4tBuMB was used as the fluorescent emitter, while 4CzIPN and 4CzTPN were selected as sensitizers for 4tBuMB, and the corresponding device architecture is shown in Figure 5 [23].

A novel red fluorescence dopant 4tBuMB was designed and synthesized for use in TADF sensitizing HF-OLED. This material exhibits a high PLQY and excellent overall performance. Its maximum emission peak in toluene solution is 620 nm, which is a very standard red OLED material. The LUMO level of the 4thbumb prepared in the experiment is relatively deep. Its PL spectrum has a significant overlap with the absorption spectra of 4CzTPN and 4CzIPN. The maximum EQE, maximum EL, FWHM, and CIE coordinates are 19.4%, 617 nm, 44 nm, and (0.64, 0.36), respectively. This is the best device performance reported for red HF-OLEDs to date. Therefore, we believe that this work will contribute to the development of efficient red hole devices.

In the field of organic luminescent materials, improving luminescent properties and optimizing energy transfer efficiency have long been central research focuses. Among them, 4tBuMB, as a material with unique properties, has an energy level structure and spectral characteristics that show special value in many applications. Based on these characteristics of 4tBuMB, the researchers carefully screened and considered two TADF materials, 4CzIPN and 4CzTPN, to sensitize it. Because of its unique luminescence mechanism, TADF materials can achieve high efficiency luminescence through reverse intersystem crossover in excited states, which provides the possibility to improve the performance of the whole system. Specifically, the two TADF materials, 4CzIPN and 4CzTPN, each have different characteristics. Among them, 4CzTPN and 4tBuMB have a larger overlap region in the spectrum. This large spectral overlap means that the emission spectrum of 4CzTPN is better matched to the absorption spectrum of 4tBuMB during energy transfer. When 4CzTPN is excited to produce energy, the emitted photons are more easily absorbed by 4tBuMB, thus promoting the effective transfer of energy. In 4CzTPN as a TADF material-sensitized 4tBuMB structure, due to the greater spectral overlap, the FRET process can be faster. This fast energy transfer enables the whole system to utilize an excited state energy more efficiently and reduces energy loss. At the same time, because more energy can be transferred to 4tBuMB, its luminous spectrum becomes narrower. Thus, higher color purity is obtained, which has important application value in display technology and other fields [71,72,73,74]. This is the best red HF-OLED device performance reported to date.

In OLEDs, researchers are working to optimize the combination of TADF materials and fluorescent emitters to achieve more efficient and accurate energy transfer. For example, in 2017, the Yasuda team proposed an innovative design in which fluorescent emitters are encapsulated by dendritic peripheral units, which helps to inhibit the energy loss of triplet excitons and the process of direct carrier capture [75]. In the same year, Duan’s research group also made a breakthrough by developing conventional fluorescent dopants suitable for vacuum evaporation processes and protected by inert end substituents with small molecular weight [10]. Both methods increase the intermolecular distance, thereby reducing unnecessary energy transfer processes and improving the performance of the device. Further studies also show that the introduction of inert groups on TADF materials is beneficial to improve device efficiency [76]. However, the sp^3^-hybrid characteristics of tert-butyl may hinder the carrier transport and subsequent recombination process, resulting in high on-voltage problems of up to 6.5 V in some high-performance fluorescence devices [74].

To enhance devices’ performance, it is critical to develop novel peripheral inert substituents and to gain a deeper understanding of the mechanisms by which they influence the energy transfer process. This can enhance the performance of the device, and reduce the operating voltage, and promote the OLED technology to a more efficient and economical direction. Due to its excellent carrier transport capacity, high triplet energy level, and significant molecular rigidity, fluorene units have been widely used in OLEDs to manufacture carrier transport materials, main materials, and components of light-emitting layers [77,78,79]. In addition, studies have found that phenyl-fluorene units with large steric hindrance are an excellent substituent, which can effectively improve the molecular level orientation, device efficiency, and stability of OLEDs [80].

**Figure 6:**
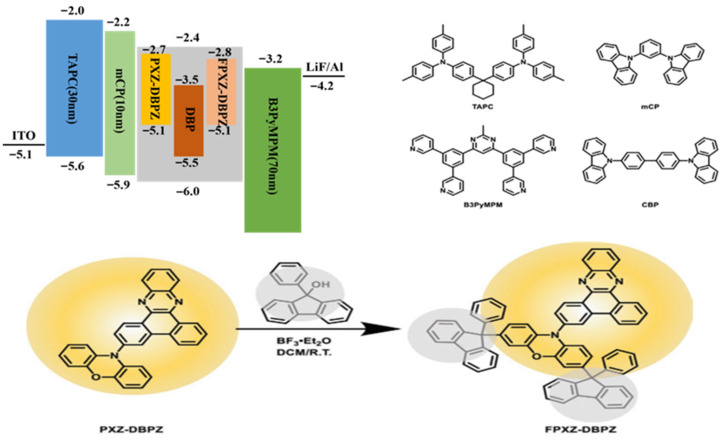
Molecular formula and device structure [24].

Based on these findings, Xie et al., in 2022, committed themselves to preparing FPXZ-DBPZ as an inert isolation unit by introducing phenyl-fluorene as a substituent on TADF assistant host PXZ-DBPZ [24,81]. The goal is to block undesirable energy transfer processes in high-fluorescence devices, especially those that result in reduced efficiency of the DET processes. With this molecular design strategy, we expect to be able to optimize the performance of the device, improve its efficiency and stability, while reducing energy loss. Its molecular formula and device structure are shown in Figure 6 [24].

Device configuration: ITO/TAPC (30 nm)/mCP (10 nm)/EMLs (20 nm)/B3PyMPM (70 nm)/LiF (1 nm)/Al. PXZ-DBPZ is used as a TADF assistant host material, and its properties have been characterized in high frequency devices. The device is composed of CBP as the host material, FPXZ-DBPZ and PXZ-DBPZ as the TADF material for comparison, and DBP as the fluorescent material, and its structure is CBP:9%FPXZ-DBPZ or PXZ-DBPZ:0.6%DBP. The experimental results show that the high frequency devices prepared on the basis of PXZ-DBPZ show low color purity, which may be caused by insufficient energy transfer. These results reveal the potential to optimize the performance of high-frequency OLED devices by tuning TADF assistant host materials, while also pointing to key factors to consider when designing efficient, high color purity devices. Considering that the law DBP doping concentration in the device, we can infer that the main route of energy loss may be through DET. In this case, the excellent performance of FPXZ-DBPZ-based high-frequency devices can be attributed to the effective blocking of DET process by terminal phenyl fluorene substituents. By reducing this energy loss pathway, phenyl-fluorene substituents help improve the efficiency of the overall device.

In this experiment, two phenyl-fluorene groups were introduced into the TADF material as peripheral inert substituents, effectively suppressing the energy loss process in hyperfluorescent OLEDs. This molecular design strategy has been verified in a ternary blended film based on FPXZ-DBPZ, where the triplet DET rate is significantly reduced. The results make the device one of the most efficient in narrow-band emitting red high-frequency OLEDs that have been reported. Our study reduces the DET energy loss process and provides a new strategy for TADF dopant design. The characteristics of the high-performance red HF-OLEDs devices reported in recent years are shown in Table 3.

With the continuous advancement of science and technology, red hyperfluorescent OLED technology is expected to be widely applied in lighting and gradually replace traditional luminous technology. This technology will not only bring users a richer and more efficient visual experience but also show great potential and broad application prospects in lighting due to its performance. However, the red organic materials are usually prone to decomposition due to their photosensitive properties. This instability can lead to reduced brightness and shortened life after prolonged use of the device. At the same time, the width of luminescence spectrum red OLEDs is wider, resulting in unsatisfactory color purity. Therefore, this will be a challenge for red OLEDs, which needs to be solved by researchers through continuous trial and innovation.

### 2.4. White Hyperfluorescent OLEDs Based on TADF-Sensitized Fluorescence

The development of white organic light-emitting diodes (WOLEDs) is crucial for achieving efficient and highly stable devices. Traditionally, white light can be produced either by combining complementary blue and yellow emissions or by mixing the three primary colors of blue, green and red. Among them, the EQE of the fluorescent material is very low, and the stability of the phosphorescent material is not good, so the scientists designed the hyperfluorescent structure, which is sensitized by the TADF material to emit fluorescence, to obtain an efficient and stable device. In 2018, Song et al. tried to introduce blue and yellow fluorescent materials into blue TADF materials and finally achieved excellent white light through TADF-sensitized fluorescent luminescence. So we can come to a conclusion, by combining TADF sensitizers with fluorescent materials, the performance of WOLEDs will be greatly improved [82].

**Figure 7:**
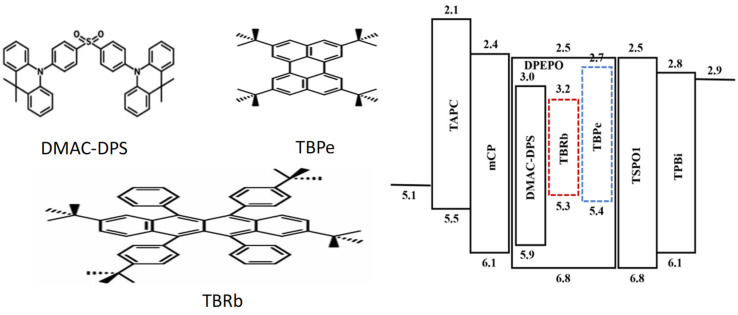
Molecular formula and device structure [82].

In this experiment, the blue TADF material DMAC-DPS was used as the sensitizer, combined with the fluorescent materials TBPe and TBRb as the emission layer, to achieve white emission. In this system, DMAC-DPS acts as a sensitizer transferring energy to TBPe and TBRb, which are ultimately illuminated by the fluorescent material, resulting in highly efficient white luminescence. This novel WOLED shows excellent performance, with color coordinates up to (0.26,0.32), presenting high-quality white light. At the same time, the device has excellent performance in photoelectric conversion efficiency. Its structure and molecular formula are shown in Figure 7 [82]. It provides important information for understanding and reproducing this innovative research. Through careful material selection and device structure design, this research not only improves the efficiency of all-fluorescent WOLEDs but also lays a solid foundation for the future development and application of high-performance OLED materials.

Owing to the sensitizing effect of blue TADF emitters, these devices exhibit higher EQE than conventional fluorescent WOLEDs. Therefore, employing the hyperfluorescent process can effectively enhance device performance.

**Figure 8:**
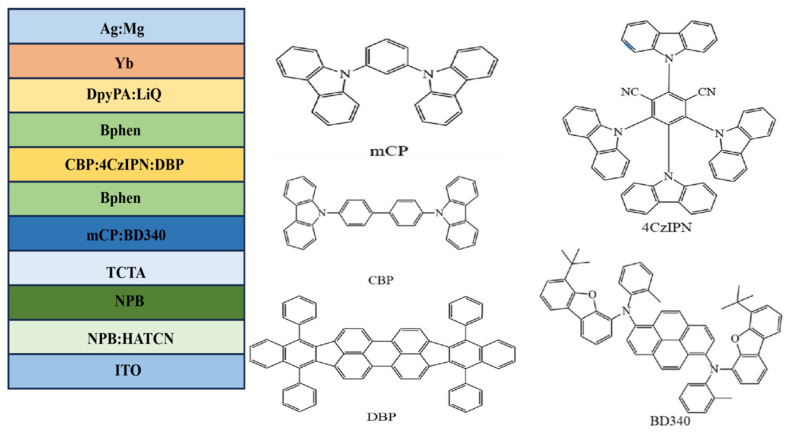
Molecular formula and device structure [83].

The use of phosphorescent and TADF materials enables efficient luminescence, as both types of materials can efficiently utilize triplet excitons, thereby reducing energy loss and achieving 100% internal quantum efficiency (IQE). However, because of its internal structure and characteristics, phosphorescent materials have a high cost and environmental pollution problems. In contrast, TADF materials do not have these concerns and are expected to replace phosphorescent materials in the future. However, TADF materials also have a serious problem of rolling down at high current density. During TADF OLED operation, because the RISC process rate is limited, it leads to the accumulation of triplet excitons. This accumulation of excitons leads to a variety of exciton annihilation processes, which is a key factor leading to efficiency rollout and shortened device life. In order to solve the above problems, in 2023, Gao et al. proposed a new OLED structure, the hyperfluorescent luminescent layer [83], to reduce this accumulation. This new structure is usually composed of the main material, TADF material, fluorescent material together to form the luminous layer of OLED, to optimize the performance of the device and extend its service life. In this structure, the TADF material acts as a sensitizer, whose role is to cause the final fluorescent emitter to emit light, which fully demonstrates the superior properties of TADF materials. At the same time, the energy can be transferred to the fluorescent material to the maximum extent, to achieve efficient luminescence. This effectively improves the problem of exciton accumulation, thereby reducing the problem of rolling down. Its structure and molecular formula are shown in Figure 8 [83].

**Figure 9:**
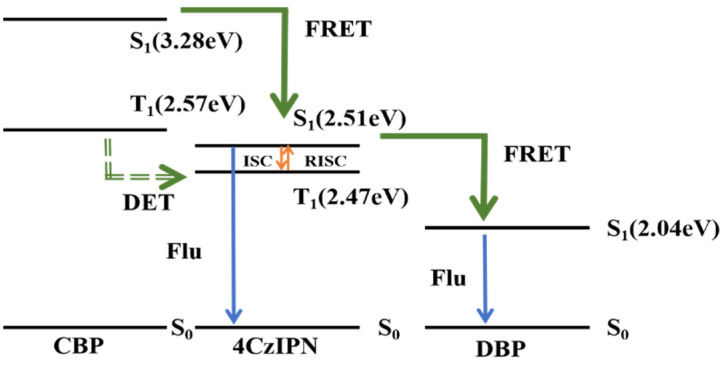
The energy transfer process of the white hyperfluorescent device [83].

The optimized device structure is ITO/NPB:HATCN (10 nm)/NPB (50 nm)/TCTA (10 nm)/mCP:BD340 (3 wt.%) (20 nm)/Bphen (4 nm)/CBP:4CzIPN (15 wt.%):DBP (0.2 wt.%) (5 nm)/Bphen (5 nm)/DpyPA:LiQ (30 nm)/Yb (1 nm)/Ag:Mg (90 nm) [83]. By combining a yellow fluorescent layer and a blue fluorescent layer, a white all-fluorescent OLED with improved efficiency can be obtained. In this experiment, it can be seen that a standard white all-fluorescent OLED is obtained according to its CIE coordinates. The maximum external quantum efficiency is 8.39%. The energy transfer process of the white hyperfluorescent device is shown in Figure 9 [83].

The figure illustrates the mechanism of hyperfluorescent OLED. Since CBP has the highest singlet and triplet energy levels, energy is transferred to the singlet and triplet states of 4CzIPN and DBP via FRET and DET processes, respectively. 4CzIPN is a TADF material, which can upconvert excitons to the singlet energy level, part of the energy can be transferred to the DBP material through FRET, and part of the energy can be used for 4CzIPN’s own luminescence. Therefore, the energy transfer is incomplete, 4CzIPN and DBP materials emit light at the same time, and finally emit yellow light. Combined with the blue light emitting layer together to produce efficient white light. An experimental design was carried out to incorporate an interlayer between the two luminescent layers to achieve complementary color luminescence. Eventually, the maximum luminance reached 9606 cd m^−2^, with the maximum CE, PE, and EQE being 22.2 cd A^−1^, 19.2 lm W^−1^, and 10.90%, respectively. The best CRI reached 96, and the Von value was reduced to 3.6 V. This proves the feasibility of this strategy and also demonstrates the universality of the hyperfluorescent luminescence system and the interlayer strategy in the field of tricolor white light OLEDs with full fluorescence emissions. This is of great help in achieving high efficiency, long lifespan, and good luminescence quality in white light devices. The characteristics of the high-performance white HF-OLEDs devices reported in recent years are shown in Table 4.

By combining the advantages of both TADF and fluorescent materials, white HF-OLEDs have emerged as a promising technology for achieving high-efficiency, high-color-purity white light emission [85]. Current research has achieved significant breakthroughs in efficiency, lifetime, and industrialization. However, challenges such as the stability of blue emitters and mass production processes still require continuous efforts to overcome [86]. Meanwhile with ongoing innovations in materials, advancements in fabrication processes, and the expansion of application scenarios, HF-WOLEDs are poised for large-scale commercialization in the display and lighting sectors, potentially reshaping the global display industry landscape [87]. Furthermore, with ongoing research and innovation, it is certain that even more advanced devices will be developed.

## 3. The Future and Prospect of Hyperfluorescent Organic Light-Emitting Diodes

Hyperfluorescent OLEDs have developed rapidly owing to their distinctive characteristics, marking a wave of technological innovation. Looking ahead, with the refined optimization of the structure and the generation of innovative materials, the power efficiency of hyperfluorescent devices is expected to achieve a leap. However, the stability of its blue emission still needs to be improved, which has become the core bottleneck limiting the commercialization of hyperfluorescent white OLEDs. At the same time, although the blue MR-TADF materials show excellent performance in color purity and efficiency, their slow triplet upconversion rate limits their stability at high brightness. Concurrently, in hyperfluorescent systems, the DET process can lead to severe efficiency roll-off and color shift. To address these issues, we can design blue MR-TADF materials with a rigid skeleton and peripheral inert groups to reduce intermolecular interactions and suppress exciton annihilation, thereby improving the chemical stability of the blue materials and extending device lifetime.

At the same time, innovative advances in large-scale production and materials science are expected to significantly reduce the cost of hyperfluorescent OLEDs, paving the way for their adoption and penetration in the consumer electronics market. From smartphone screens to home lighting systems, the application landscape of hyperfluorescent OLEDs will expand dramatically and become an indispensable part of daily life.

The future development prospects of HF-OLEDs are highly promising, as they not only show great potential in addressing the stability and color purity challenges of existing OLED technologies, but also as the key role in high-performance devices. Hyperfluorescent OLEDs will undoubtedly set off a far-reaching change in the organic device industry.

## 4. Conclusions

In recent years, hyperfluorescent OLEDs have opened pathways toward achieving highly efficient OLEDs with high color purity by combining the RISC capability of triplet excitons in TADF materials with the extremely narrow emission spectra of fluorescent emitters. Taking hyperfluorescent OLEDs as a starting point, this paper reviews recent advances in hyperfluorescent materials, device architectures, and related technologies both domestically and internationally. The emission mechanisms and performance optimization strategies of hyperfluorescent OLEDs are systematically summarized from the perspectives of blue, green, red, and white emitters, thereby providing a valuable reference for the future development of this field.

## Figures and Tables

**Table 1:** The characteristics of the reported high-performance blue HF-OLEDs devices.

Emitters	CE (cd A^−1^) ^a^	PE (lm W^−1^) ^b^	EQE (%) ^c^	FWHM (nm) ^d^	CIE ^d^	Ref
v-DABNA	39.0/31.0	41.0/16.0	27.0/20.0	18.0	(0.15,0.20)	[18]
v-DABNA	44.5/40.4	-/-	37.7/32.6	21.0	(0.13,0.22)	[26]
v-DABNA	42.0/36.6	-/-	43.9/37.5	21.0	(0.12,0.26)	[26]
v-DABNA	37.0/33.0	36.0/31.0	27.3/18.6	17.7	(013,0.17)	[14]
p3IDCz	38.0/26.1	-/-	30.7/21.8	16.0	(0.13,0.16)	[27]
P3IDCz	48.5/30.8	-/-	36.4/23.1	16.0	(0.12,0.19)	[27]

^a^ Current efficiency maximum value, value at 1000 cd m^−2^; ^b^ power efficiency maximum value, value at 1000 cd m^−2^; ^c^ EQE maximum value, EQE value at 1000 cd m^−2^; ^d^ CIE and FWHM at 1000 cd m^−2^.

**Table 3:** The characteristics of the reported high-performance red HF-OLED devices.

Emitters	CE (cd A^−1^) ^a^	PE (lm W^−1^) ^b^	EQE (%) ^c^	FWHM (nm) ^d^	CIE ^d^	Ref
4tBuMB	26.0/24.0	-/-	19.4/17.2	44.0	(0.64,0.36)	[23]
4tBuMB	21.1/20.1	-/-	13.3/11.9	48.0	(0.57,0.40)	[23]
DBP	28.1/-	26.0/-	18.1/-	57.9	(0.61,0.38)	[24]
DBP	29.6/-	24.5/-	15.2/-	38.0	(0.57,0.42)	[24]
DBP	25.0/20.0	28.0/10.0	17.5/10.9	-	(0.61,0.39)	[5]
DBP	22.1/-	27.8/1.8	16.9/2.6	-	(0.65,0.35)	[73]

^a^ Current efficiency maximum value, value at 1000 cd m^−2^; ^b^ power efficiency maximum value, value at 1000 cd m^−2^; ^c^ EQE maximum value, EQE value at 1000 cd m^−2^; ^d^ CIE and FWHM at 1000 cd m^−2^.

**Table 4:** The characteristics of the reported high-performance white HF-OLEDs devices.

Emitters	CE (cd A^−1^) ^a^	PE (lm W^−1^) ^b^	EQE (%) ^c^	FWHM (nm) ^d^	CIE ^d^	Ref
TBRb/TBPe	29.8/24.0	31.1/17.3	12.9/10.8	-	(0.26,0.32)	[82]
TBRb/TBPe	31.1/26.1	31.6/17.8	11.2/9.7	-	(0.35,0.40)	[82]
4CzIPN/DBP/BD340	16.2/13.7	13.1/6.6	9.0/7.5	-	(0.44,0.40)	[83]
4CzIPN/DBP/BD340	22.2/8.8	19.2/4.3	10.9/4.3	-	(0.34,0.37)	[83]
TFP/PTZTPE-3	-/-	-/-	8.2/-	-	(0.28,0.38)	[84]

^a^ Current efficiency maximum value, value at 1000 cd m^−2^; ^b^ power efficiency maximum value, value at 1000 cd m^−2^; ^c^ EQE maximum value, EQE value at 1000 cd m^−2^; ^d^ CIE and FWHM at 1000 cd m^−2^.

## Data Availability

No new data were created or analyzed in this study. Data sharing is not applicable to this article.
